# The Alcohol Hangover Research Group: Ten Years of Progress in Research on the Causes, Consequences, and Treatment of the Alcohol Hangover

**DOI:** 10.3390/jcm9113670

**Published:** 2020-11-16

**Authors:** Joris C. Verster, Lizanne Arnoldy, Sarah Benson, Andrew Scholey, Ann-Kathrin Stock

**Affiliations:** 1 Division of Pharmacology, Utrecht Institute for Pharmaceutical Sciences (UIPS), Utrecht University, 3584CG Utrecht, The Netherlands; larnoldy@swin.edu.au; 2 Centre for Human Psychopharmacology, Swinburne University, Melbourne VIC 3122, Australia; sarahmichellebenson@gmail.com (S.B.); andrew@scholeylab.com (A.S.); 3 Cognitive Neurophysiology, Department of Child and Adolescent Psychiatry, Faculty of Medicine, TU Dresden, Fetscherstr. 74, 01,307 Dresden, Germany; Ann-Kathrin.Stock@uniklinikum-dresden.de; 4 Biopsychology, Department of Psychology, School of Science, TU Dresden, Zellescher Weg 19, 01,069 Dresden, Germany

**Keywords:** alcohol, hangover, causes, consequences, treatments

## Abstract

The alcohol hangover is defined as the combination of negative mental and physical symptoms, which can be experienced after a single episode of alcohol consumption, starting when blood alcohol concentration (BAC) approaches zero. Here, we present the book “The alcohol hangover: causes, consequences, and treatment”, written to celebrate the 10th anniversary of the Alcohol Hangover Research Group (AHRG), summarizing recent advances in the field of alcohol hangover research.

The alcohol hangover is defined as the combination of negative mental and physical symptoms, which can be experienced after a single episode of alcohol consumption, starting when blood alcohol concentration (BAC) approaches zero [1,2]. Despite the fact that the alcohol hangover is the most commonly reported negative consequence of alcohol consumption [3], a relatively small amount of research has been devoted to this topic. The latter is surprising as the alcohol hangover is associated with negative mood, cognitive impairment, and physical effects [4]. Here, we present the book “The alcohol hangover: causes, consequences, and treatment”, written to celebrate the 10th anniversary of the Alcohol Hangover Research Group (AHRG).

In 2010, the AHRG was founded to raise the profile of alcohol hangover research [5]. The AHRG scientific meetings aim to bring together active and internationally diverse alcohol hangover researchers to generate discussion on recent developments in hangover research. The objectives of these meetings are to discuss recent findings and future research directions, to raise the profile of alcohol hangover research, and to start new research collaborations. Over the past 10 years, 11 successful AHRG meetings have been held across the world [5,6,7,8,9,10,11]. In 2010, the first AHRG meeting was held as a satellite symposium of the Research Society on Alcoholism conference in San Antonio, Texas, USA. Subsequent AHRG meetings were held in Paris in 2010 (France), Utrecht in 2011 (The Netherlands), Wolfville in 2012 (Canada), Keele in 2013 (UK), Bellevue in 2014 (USA), Perth in 2015 (Australia), New Orleans in 2016 (USA), Utrecht in 2017 (The Netherlands), Utrecht in 2018 (The Netherlands), and Wailoaloa Beach, Nadi in 2019 (Fiji). Proceedings of most of the AHRG meetings have been published [5,6,7,8,9,10,11].

In the decade since its inception, the AHRG has moved the field forward significantly. The inaugural meeting resulted in the publication of a consensus paper on best practice in hangover research, and an evaluation of the gaps in knowledge that should be addressed by future research [12]. Among the key accomplishments was the development of a definition for the alcohol hangover [1]. In addition, international research collaborations resulted in a significant increase in the number of published articles on the alcohol hangover (see Figure 1).

In conjunction with its 10th anniversary, a Special Issue of the Journal of Clinical Medicine on the alcohol hangover was curated by members of the AHRG. After peer-review, twenty-five articles were accepted for the Special Issue, and this collection is combined in this book, entitled “The alcohol hangover: causes, consequences, and treatment”.

The first five articles discuss significant methodological advances. In the first article, an update of the definition of the alcohol hangover is discussed [2]. The update of the definition was necessary, as current thinking about the traditional threshold for experiencing hangovers (a BAC of at least 0.11%) had changed. The new consensus, which is discussed in article two, reflects observations that hangovers can be experienced at any BAC [13].

Article three discusses the advantages and limitations of naturalistic study designs and their implementation in alcohol hangover research [14]. In contrast to traditional, controlled clinical trials, hangover research often applies a naturalistic study design in which investigators do not interfere with the drinking session. The article explains why this is important in terms of ecological validity (i.e., a real-life drinking session at a venue of choice, with corresponding behaviors and real-life alcohol consumption levels), and to what extent the naturalistic design has an impact on reliability and validity of study outcomes in comparison to highly controlled clinical trials. Article four discusses the assessment of overall hangover severity [15]. Traditional research has used composite symptom scales to assess hangover severity. The advantage of this approach is that information is gathered about the presence and severity of individual hangover symptoms. However, the research discussed in article four demonstrates that there are several disadvantages to this approach (e.g., the choice of included individual symptoms in a scale determines the overall hangover severity score, which therefore differs between currently used scales). The findings suggest that a one-item hangover severity assessment has advantages over composite symptom scale scores. In the final part of this section, article five discusses the prevalence of hangover resistance according to two methods for calculating estimated BAC [16]. The findings discussed show that different equations used to calculate estimated BAC yield different outcomes. The latter is an important finding, and future consensus is warranted among AHRG members to ensure harmonization in reporting estimated BAC to allow direct comparisons of research from different groups.

The next two articles discuss the “causes” of alcohol hangover, and articles review the current knowledge on the pathology of the alcohol hangover. Whereas previous reviews on the causes of the alcohol hangover relied heavily on research data from the 1970s by the Finnish group Ylikhari et al., [17,18,19], the articles in this book provide major advances in the understanding of the pathology of the alcohol hangover. Article six reviews the role of alcohol metabolism in the pathology of the alcohol hangover [20], and article seven presents new data on the inflammatory response to alcohol consumption and its contribution to the alcohol hangover [21]. The data reveal that the rate of ethanol metabolism is an important predictor of next day hangover severity. In addition, the impact of oxidative stress and the balance between free radicals and antioxidants is discussed, as well as the role of acetaldehyde in eliciting an inflammatory response to alcohol (e.g., the release of cytokines), which ultimately elicits the alcohol hangover.

The following six articles discussing a variety of factors (‘correlates’) that may exacerbate or attenuate hangover symptoms. Article eight presents data on the effect of dietary nutrient intake on alcohol hangover severity [22]. Dietary nutrients are frequently included as ingredients in hangover treatments. Therefore, it is of interest to verify which of these, taken as part of daily diet, are associated with experiencing less severe hangovers. The results indicate that drinkers who consume food rich in zinc and nicotinic acid report less severe hangovers. Both nutrients are involved in the breakdown of ethanol and acetaldehyde, which may explain these findings. Article nine discusses the fact that different drinking levels are associated with experiencing differential levels of hangover severity [23]. The data confirm previous findings that hangover symptom severity is most severe among heavy and chronic drinkers. Article 10 discusses the interesting finding that when individuals experience hangovers more frequently, their severity increases [24]. Contrary to the common notion that drinkers get used to the amount of alcohol they consume and become “immune” to the adverse effects of drinking at this level, this observation suggests that reverse tolerance develops. Article 11 discusses the finding that hangover symptom severity is to some extent determined by the level of pain catastrophizing of drinkers [25]. Reporting higher levels of pain catastrophizing, in particular rumination, was associated with experiencing more severe hangovers. This finding is important, as it may have implications for the percentage of drinkers reporting being hangover resistant, and illustrates that the psychological perception of “what is pain?” and “what is mild, moderate, or severe?” differs between individuals, and thus impacts the reporting of the presence and rating of the severity of hangover symptoms. The latter is important as to date, no objective assessments for alcohol hangover (symptom) severity are available, and researchers have to rely on subjective reporting. Article 12 reviews possible sex differences in the presence and severity of hangover symptoms [26]. In contrast to acute alcohol effects (e.g., greater ratings of subjective intoxication in women), sex differences in the next-day effects of alcohol consumption appear to be limited. Finally, article 13 discusses the impact of mood and subjective intoxication on hangover severity [27]. Whereas baseline mood and mood while drinking had no relevant impact on next-day hangover severity, subjective intoxication (i.e., the level of drunkenness) showed to be a strong determinant of hangover severity.

Eleven subsequent articles discuss various aspects of cognitive, psychomotor, and physical performance during the hangover state (‘consequences’). Article 14 describes the results of a study that assessed cognitive functioning and mood, applying a naturalistic study design [28]. The study demonstrates that participants can be tested at home using mobile technology to collect data. This methodology has clear advantages for participants (they do not have to come to the research center) and logistics for researchers (no lab space needed). Article 15 describes an investigation in which participants were approached on premise after consuming alcohol [29]. Both objective (breathalyzer) and subjective assessments (interview) of alcohol consumption and intoxication were made, and subjects were invited to complete online assessments of hangover severity and cognitive performance the next morning. Articles 14 and 15 demonstrate the utility of mobile/online assessments for hangover research. Future direct comparisons should investigate if validity and reliability of at home testing are equal to that of testing in controlled laboratory environments. Driving a car is one of the common daily activities that are potentially dangerous, as the use of alcohol can significantly impair driving performance and increase the chances of having an accident. Numerous studies have demonstrated that driving a car or bicycle while intoxicated is unsafe [30,31]. Previous research has also shown that simulated highway driving whilst experiencing a hangover is significantly impaired [32]. Despite this knowledge, a substantial number of both private and professional drivers continue to drive a car while experiencing a hangover [33]. Article 16 discusses the impact of alcohol hangover on simulated driving performance during a “commute to work” [34]. The study also revealed that during a relatively short drive, driving performance was significantly impaired while hungover. Driving is also a clear example of multitasking. In article 17, Benson et al. investigate alcohol hangover effects on another behavior, which can be translated to everyday workload, namely multitasking [35]. A hangover was associated with worse mood (reduced alertness and contentment, and increased anxiety and mental fatigue), and poorer multitasking performance, with greater effort needed to complete the tasks compared to the non-hangover condition. Interestingly, stress reactivity was not differentially affected by the hangover. The effects of alcohol hangover on executive functions are discussed in article 18. The investigation by Gunn et al. found that the alcohol hangover impairs core executive function processes that are important for everyday behaviors, such as decision-making and planning [36].

Affected daily behaviors and cognitive functioning during the hangover may ultimately be related to impaired information processing during the hangover. Three articles from Stock and colleagues investigated this in more detail. In article 19, they demonstrate that the alcohol hangover differentially modulates the processing of relevant and irrelevant information [37], and article 20 discusses findings showing that the alcohol hangover slightly impairs response selection but not response inhibition [38]. Finally, article 21 shows that the alcohol hangover does not alter the application of model-based and model-free learning strategies [39]. Together, these three articles provide further insights into the nature of slowed and impaired information processing during a hangover.

Whereas much research is devoted to cognitive aspects of the alcohol hangover, our understanding of the effects on physical state and sports performance has been limited. In article 22, Devenney et al. [40] report on physical activity level assessments comparing hangover and alcohol-free days. Using mobile technology, the continuous assessments of activity levels showed that during hangover subjects performed at lower activity levels, and vigorous activities were absent. Additionally, the assessments revealed that sleep quality was significantly poorer after the evening of alcohol consumption. Article 23 discusses the results of a study investigating the effects of alcohol consumption and hangover on endurance performance. Subjects on holiday walking the 18 km Samariá Gorge on the island of Crete in Greece were surveyed before and after they completed the walk. The analysis revealed that a variety of factors may predict walking performance and effort required to perform the walk, ranging from baseline physical state, immune fitness, to past night sleep quality, and also alcohol consumption and hangover severity [41].

Two articles discuss the outcomes of recent clinical trials that evaluated potential new hangover treatments. Despite a clear demand from drinkers who experience hangovers [42], currently there are no hangover treatments where the effectiveness has been demonstrated in independent double-blind, placebo-controlled clinical trials [43,44,45]. The increasing knowledge on the pathology of the alcohol hangover has resulted in focusing treatment development on products that aim to reduce the inflammatory response to alcohol and/or to enhance alcohol metabolism. In article 24, results of a pilot study are presented examining the effectiveness of SJP-005, a combination product of naproxen and fexofenadine, aiming to prevent hangovers by reducing the inflammatory response to alcohol consumption [46]. In article 25, the effects of Rapid Recovery are discussed—a hangover treatment aiming to reduce oxidative stress—and thereby preventing hangovers or reducing their severity [47].

Taken together, “The alcohol hangover: causes, consequences, and treatment” provides a comprehensive overview of current insights and research into many aspects of the alcohol hangover. The book highlights the advances in the field over the past decade, fueled by successful collaborations of researchers of the Alcohol Hangover Research Group and others investigating the interesting yet sometimes puzzling phenomenon of the alcohol hangover.

## Figures and Tables

**Figure 1:**
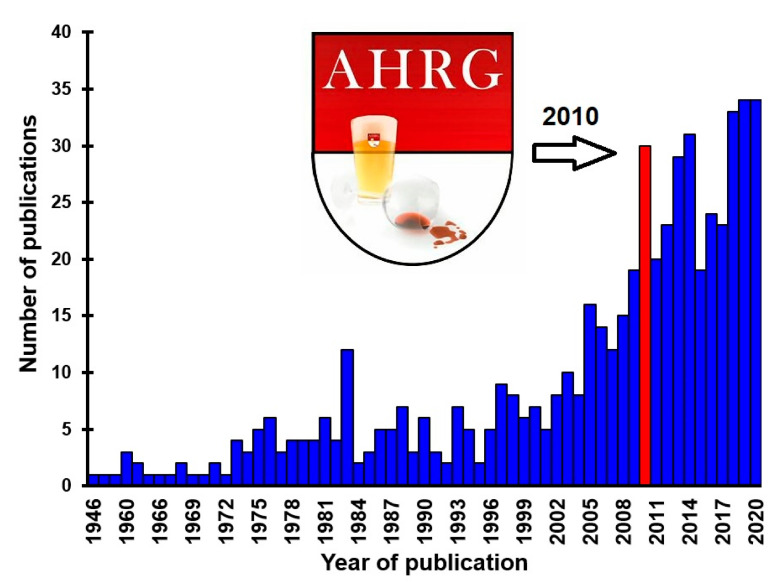
Published articles on the alcohol hangover. Number of publications taken from PubMed (https://pubmed.ncbi.nlm.nih.gov, assessed on 13 October 2020), searching for “alcohol hangover”. In 2010 (red bar), the Alcohol Hangover Research Group (AHRG) was founded.
